# Intelligent therapeutic robotic-assisted surgery as the next frontier of precision oncology

**DOI:** 10.3389/fonc.2026.1825904

**Published:** 2026-05-20

**Authors:** Shakta Mani Satyam, Adil Farooq Wali, Mohamed El-Tanani, Ali Sarfraz, Ibrahim Khalil, Wasim Iyad Alghoul, Areebah Ajaz Bhat, Iqra Mansor, Mohammed Abdulla Alhammadi, Wed Burhan Jameel Al-Shammari, Rashed Ali Salem Nasser, Aiswarya Menon, Omar Mohamed Alahmad

**Affiliations:** 1 Department of Pharmacology, Ras Al Khaimah (RAK) College of Medical Sciences, Ras Al Khaimah (RAK) Medical and Health Sciences University, Ras Al Khaimah, United Arab Emirates; 2 Ras Al Khaimah (RAK) College of Pharmacy, Ras Al Khaimah (RAK) Medical and Health Sciences University, Ras Al Khaimah, United Arab Emirates; 3 Department of Biotechnology, American University of Ras Al Khaimah, Ras Al Khaimah, United Arab Emirates

**Keywords:** artificial intelligence in surgery, intelligent surgical systems, intraoperative drug delivery, pharmacological robotics, precision cancer therapeutics, robotic oncology

## Abstract

Robotic-assisted surgery has evolved over the past two decades from mechanically assisted laparoscopy into an increasingly intelligent, biologically responsive therapeutic modality. While early robotic systems primarily enhanced dexterity and visualization, contemporary platforms now integrate artificial intelligence, advanced imaging, real-time biosensing, and pharmacological interfaces, extending surgical capability beyond physical manipulation alone. Robotic-assisted surgery is thus emerging at the intersection of digital intelligence and molecular medicine, with the potential to sense, interpret, and modulate tumor biology during operative intervention. However, current literature remains largely procedure-centered and mechanically focused, offering limited synthesis of how robotic systems converge with pharmacology, artificial intelligence, and systems medicine to actively regulate oncologic processes. A cohesive, oncology-oriented framework integrating intelligent robotics with intraoperative drug delivery and real-time biological feedback is notably lacking. This narrative review critically consolidates recent advances in pharmacologically enabled robotic platforms, AI-driven intraoperative cognition, and emerging closed-loop surgical–pharmacological architectures. It examines next-generation systems incorporating localized drug delivery, molecular diagnostics, and real-time pharmacodynamic monitoring, while discussing translational pathways and regulatory considerations shaping semi-autonomous oncologic ecosystems. By reframing robotic-assisted surgery as a dynamic therapeutic science rather than a purely mechanical tool, this review highlights a new frontier in precision oncology that has the potential to enable individualized, adaptive, and predictive cancer interventions.

## Graphical Abstract

**Figure:**
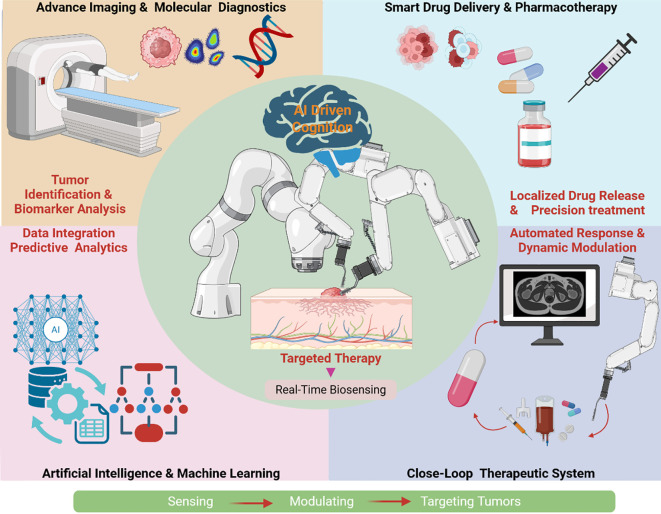
This graphical abstract was Created in BioRender. Wali, A. F. (2026), https://BioRender.com/86hr60b by one of the co-authors using a personal BioRender license.

## 1 Introduction

Robotic-assisted surgery stands as one of the most transformative technological advances in modern medicine, reshaping not only how surgical procedures are performed but also how operative interventions are conceptualized within the broader therapeutic landscape. “Robotic-assisted surgery (RAS)” refers to currently established clinical systems primarily designed for enhanced dexterity and visualization. The earliest robotic systems emerged to address the ergonomic and technical limitations of conventional laparoscopy by enhancing instrument articulation, motion scaling, and visualization (1). These innovations significantly expanded the scope of minimally invasive surgery and enabled levels of precision previously unattainable through manual techniques (2, 3). However, the contemporary trajectory of robotic-assisted surgery has moved well beyond dexterity enhancement. Robotic-assisted surgery is now entering a decisive new era in which it is no longer defined by enhanced dexterity or minimally invasive access, but by its emerging capacity to function as an intelligent, biologically responsive, and pharmacologically empowered therapeutic platform (4). Modern surgical platforms are increasingly embedded within digital, computational, and biological networks, redefining the surgeon’s role from manual operator to cognitive supervisor of adaptive cyber–physical oncologic systems (5, 6).

In parallel, biomedical science has entered an era defined by systems biology, precision oncology, and data-driven therapeutics. Advances in molecular imaging, pharmacogenomics, real-time biosensing, and artificial intelligence have fundamentally altered understanding of disease heterogeneity, therapeutic responsiveness, and tumor ecosystem dynamics (7, 8). Surgical intervention is increasingly recognized not as a purely mechanical event but as a biologically active and regulatable process that influences inflammatory cascades, immune surveillance, metabolic reprogramming, tumor microenvironments, and regenerative signaling. This recognition has reframed surgical oncology from a discipline centered on structural tumor excision toward one focused on intraoperative disease engineering and biological modulation. Robotic platforms are uniquely positioned at the interface between digital intelligence and living tissue, enabling them not only to manipulate anatomy but also to sense, interpret, and actively regulate tumor biology in real time (9, 10).

The biological complexity of cancer further underscores the relevance of intelligent robotic systems. Cancer cells exist within highly heterogeneous spatial architectures, display profound molecular diversity, dynamically interact with surrounding stromal and immune compartments, and rapidly develop therapeutic resistance (11, 12). These characteristics render cancer uniquely suited for intervention by robotic platforms capable of integrating molecular imaging, and biosensing. Contemporary systems already allow surgeons to intraoperatively map metabolic gradients, immune organization, hypoxic niches, and pharmacologic responses- biological dimensions that remain inaccessible to conventional surgical approaches. As a result, the focus of robotic oncology is shifting from margin-based resection toward biologically informed intervention, in which surgery serves as a precise interface for controlling tumor ecosystems rather than merely extracting tissue.

From a pharmacological perspective, this convergence is particularly consequential. Conventional pharmacotherapy remains constrained by systemic administration, off-target toxicity, and unpredictable intratumoral drug distribution. Pharmacologically enabled robotic-assisted surgery represents the most disruptive frontier of this transformation (13). The integration of micro-injectors, drug-eluting instruments, implantable delivery systems, and theranostic probes into robotic platforms transforms surgery into a spatially programmable drug-delivery science. These technologies enable highly localized delivery of chemotherapeutics, immunomodulators, gene therapies, metabolic regulators, and regenerative signals directly into tumor and peritumoral compartments. Robotic systems have the potential to allow real-time optimization of drug dose, distribution, and biological effect; however, such capabilities are currently largely confined to preclinical investigations and early translational studies rather than routine clinical practice (4, 14).

Central to this paradigm is artificial intelligence, which functions as the cognitive engine of intelligent therapeutic robotic-assisted surgery (15). “Intelligent therapeutic robotic-assisted surgery” denotes emerging integrative platforms incorporating artificial intelligence, biosensing, and pharmacological interfaces. AI-driven perception systems enable tissue intelligence, molecular pattern recognition, and predictive modeling that exceed human sensory and cognitive limits. By integrating deep learning, reinforcement learning, and digital-twin architectures, robotic platforms can predict tumor behavior, model drug diffusion, and anticipate immune responses, dynamically modifying surgical and pharmacologic strategies during the operation (16). These capabilities establish the foundation for semi-autonomous and ultimately closed-loop oncologic surgery, in which biological signals continuously regulate both surgical actions and drug administration.

Current evidence suggests that AI-driven intraoperative cognition demonstrates promising technical feasibility in image recognition and workflow optimization; however, its translation into consistent clinical benefit remains limited by small sample sizes, algorithmic variability, lack of external validation, and heterogeneity in surgical environments. Furthermore, most available data are derived from controlled experimental or retrospective datasets rather than prospective multicenter trials, thereby limiting generalizability and clinical adoption.

Closed-loop surgery refers to an advanced surgical paradigm in which real-time biological, physiological, or molecular feedback acquired through integrated sensing technologies—is continuously processed by computational systems (including artificial intelligence) to dynamically guide and adjust surgical actions and/or intraoperative therapeutic interventions. In this framework, surgical execution and therapeutic delivery operate within a feedback-controlled system, enabling adaptive, responsive, and potentially autonomous modulation of tissue manipulation and treatment based on evolving intraoperative conditions. In this model, the operating room may potentially evolve into an emerging, real-time control environment for tumor biology, representing an early-stage conceptual framework that aims to shift from episodic treatment paradigms toward more continuous and adaptive regulation, although current evidence remains largely preclinical and requires further clinical validation.

While closed-loop surgical systems conceptually enable dynamic feedback-driven therapeutic modulation, current implementations remain largely confined to preclinical prototypes and simulation environments. Limitations include latency in real-time data integration, variability in biological signal interpretation, and absence of standardized validation frameworks. Importantly, technical feasibility does not yet equate to demonstrated clinical benefit, underscoring the need for prospective validation.

Despite these advances, current scholarship remains fragmented across engineering innovation, procedural technique, and device performance metrics. What remains conspicuously absent is a cohesive oncology-centered framework that defines robotic-assisted surgery as an intelligent, pharmacologically active therapeutic system capable of intraoperative biological control. Moreover, the clinical translation of such systems demands new regulatory and developmental paradigms that extend beyond conventional medical device evaluation. Future frameworks must accommodate algorithmic decision-making, combination drug–device therapeutics, adaptive dosing logic, and real-time pharmacovigilance, necessitating close collaboration among surgeons, pharmacologists, engineers, data scientists, and regulatory bodies.

Accordingly, this narrative review aims to establish an integrative conceptual and translational framework for intelligent therapeutic robotic-assisted surgery in oncology. To enhance scientific rigor and prevent overinterpretation, the technologies discussed in this review are explicitly stratified into three categories based on their level of evidence and translational maturity: (i) established clinical capabilities currently deployed in routine surgical oncology practice, (ii) translational and preclinical innovations supported by experimental models, early-phase clinical studies, or prototype systems, and (iii) future or conceptual frameworks that remain hypothetical and require further validation. This structured distinction is consistently applied throughout the manuscript to ensure accurate interpretation of technological readiness and clinical applicability.

## 2 Literature search strategy

This review was conducted using a structured narrative approach to ensure comprehensive coverage and methodological transparency. A literature search was performed in PubMed and Scopus databases for publications up to December 2025 using representative Boolean combinations of keywords related to robotic-assisted surgery, artificial intelligence, oncology, intraoperative drug delivery, and precision medicine. Additional sources, including regulatory documents and relevant technological reports, were identified through targeted searches. Eligible studies included peer-reviewed original research, systematic reviews, meta-analyses, and selected high-quality translational and preclinical studies relevant to the scope of robotic and AI-integrated oncologic interventions. Publications not directly related to the topic, non-evidence-based opinion articles, and redundant sources were excluded. Where overlapping literature existed, priority was given to the most recent and methodologically robust evidence, with cross-referencing to primary studies to minimize duplication bias. The retrieved literature was synthesized thematically and organized into clinically established applications, translational developments, and conceptual frameworks to support a stratified interpretation of evidence. While structured search and selection principles were applied, a formal PRISMA protocol was not adopted, as the objective of this work is to provide an integrative, forward-looking narrative synthesis across heterogeneous evidence domains rather than a quantitative systematic review.

## 3 Evolution of robotic-assisted surgery beyond conventional telemanipulation

The first robotic surgical systems introduced a telemanipulation system which used master-slave control to let surgeons perform operations through console-based controls that converted their movements into precise actions at the surgical area (17–19). The model provided exceptional stability and motion scaling and three-dimensional visualization which led to robotic platform adoption in urology and gynecology and cardiothoracic surgery and general surgery. The systems achieved their goals, but they operated as mechanical tools which extended surgeon capabilities without achieving sensory independence or intelligent adaptation or biological environment perception.

The last ten years have brought about a fundamental change from traditional surgical methods to modern platforms which combine digital technology with cognitive enhancement for surgical procedures. Modern systems focus on developing systems which combine modular construction with interoperability and small size and digital platforms that support easy integration of sophisticated imaging technology and navigation systems and data processing capabilities (20, 21). The platforms now connect to hospital information systems and imaging databases and cloud-based computational resources which support preoperative planning and intraoperative decision support and postoperative performance optimization. The operating theater now functions as a cyber-physical space which is being investigated to enable robotic-assisted surgery to operate within a connected environment where tissue and instruments and information systems interact continuously (22–24).

The development of new technology has the potential to enable robots to perform tasks which traditional laparoscopic systems used to limit. Modern soft robotic systems duplicate biological flexibility through their design which has the potential to enable safe exploration of sensitive body areas including blood vessels and brain connections and lung tissues. The surgical area becomes more accessible using magnetically operated micro-robots and endoluminal platforms which allow doctors to perform procedures in areas that standard surgical instruments cannot access. The new developments enable doctors to treat more patients while surgeons can now perform treatments at the cellular and molecular levels which unite surgical procedures with drug delivery methods.

Machine vision systems operate with machine learning technology to develop robotic perception systems (25). Modern healthcare systems now use deep learning algorithms to perform four main tasks which include anatomical landmark detection and tissue identification and blood flow evaluation and risk assessment for medical procedures (26, 27). The system facilitates robotic platforms to move from being simple tools into active surgical cognition participants (28). The development of these perceptual systems may allow systems to execute autonomous operations based on context while they modify their actions according to body state changes.

The development of new medical technologies created conditions which will enable pharmacological integration to become possible. Robotic systems now possess the ability to detect biochemical signals and measure tissue reactions and simulate biological systems which enable them to deliver therapeutic agents through exact spatial and temporal control. The development of robotic-assisted surgery represents a translational process which established robotics as the main link between surgical machinery and molecular medical practices. Pharmacologically integrated robotic platforms have demonstrated feasibility in localized drug delivery and controlled release systems in preclinical models; however, challenges related to pharmacokinetic variability, tissue penetration, regulatory approval, and safety profiling remain significant barriers to clinical translation (29–31).

The stratification presented in **Table 1** is supported by an evolving body of interdisciplinary evidence spanning robotic-assisted surgery, artificial intelligence, biosensing, and pharmacological integration. Established robotic-assisted surgical systems have demonstrated consistent clinical benefits across multiple specialties, including improved precision, reduced intraoperative blood loss, and enhanced postoperative recovery, as reported in large clinical cohorts and systematic analyses (32–35). In contrast, AI-assisted intraoperative decision-making and computer vision-guided tissue recognition are supported primarily by translational studies and early-phase clinical investigations, which demonstrate promising improvements in surgical navigation and workflow optimization but remain limited by variability in dataset quality, lack of standardization, and insufficient multicenter validation (36–40). Similarly, pharmacologically integrated robotic platforms and real-time molecular sensing technologies are currently supported primarily by preclinical and early translational studies, where feasibility has been demonstrated under controlled experimental conditions; however, robust clinical validation in human settings remains limited (41–43). Robotic systems are increasingly integrated with molecular sensing technologies (Translational evidence), while more advanced adaptive and autonomous systems remain within the domain of conceptual frameworks (Conceptual framework). More advanced paradigms, including closed-loop surgical systems, semi-autonomous robotic-assisted surgery, and bio-responsive therapeutic platforms, remain largely conceptual, supported by theoretical models and early prototype systems rather than human clinical data (26, 44–47). Collectively, this body of evidence highlights a clear gradient from clinically established technologies to emerging and conceptual innovations, underscoring the need for rigorous validation, standardized evaluation frameworks, and translational research to bridge the gap between technological potential and clinical implementation.

**Table 1:** Stratified overview of robotic-assisted surgery technologies across evidence level translational readiness and clinical applicability.

Technology/concept	Mechanistic domain	Level of evidence	Translational readiness	Current clinical applicability	Demonstrated clinical benefit	Key limitations/barriers	Regulatory and implementation challenges	Future research priorities
Robotic-assisted surgery (RAS)	Mechanical augmentation, visualization	Established clinical application	High	Widely adopted across surgical oncology, urology, gynecology	Improved precision, reduced blood loss, faster recovery	Limited biological integration; operator dependency	Device standardization; cost and accessibility	Integration with AI and biosensing systems
AI-assisted intraoperative decision support	Machine learning, predictive analytics	Translational evidence	Moderate	Limited deployment in specialized centers	Improved intraoperative guidance (early evidence)	Lack of generalizability; dataset bias	Algorithm validation; explainability requirements	Multicenter trials; real-world validation
Computer vision-guided tissue recognition	Image processing, deep learning	Translational evidence	Moderate	Pilot clinical use	Improved anatomical identification (experimental)	Variability in imaging conditions; false positives	Regulatory approval of AI imaging tools	Standardized datasets; robustness validation
Real-time intraoperative molecular sensing	Biosensing, biochemical signal detection	Translational evidence	Low–Moderate	Limited experimental and intraoperative research use	Early feasibility demonstrated	Signal instability; integration complexity	Device-drug combination regulation	Sensor miniaturization; real-time calibration
Closed-loop surgical systems	Feedback control systems, AI integration	Conceptual framework	Low	Not clinically implemented	Not established	Lack of human trials; system complexity	Safety validation; medico-legal accountability	Development of adaptive control algorithms
Pharmacologically integrated robotic platforms	Drug delivery, PK/PD modeling	Translational evidence	Low–Moderate	Preclinical and early translational studies	Localized drug delivery potential	Drug distribution variability; targeting precision	Combination product approval pathways	Controlled-release systems; targeted delivery
Multimodal data integration systems	Data fusion, systems biology	Translational evidence	Moderate	Emerging research applications	Enhanced decision support (experimental)	Data heterogeneity; computational burden	Interoperability; data governance	Real-time integration frameworks
Semi-autonomous robotic-assisted surgery	AI autonomy, robotics control	Conceptual framework	Low	Not clinically approved	Not established	Ethical concerns; safety validation	Regulatory and liability challenges	Human-AI collaboration models
Digital twin surgical modeling	Computational modeling, simulation	Conceptual framework	Low	Early-stage research	Predictive modeling potential	High computational demand; validation gaps	Integration into clinical workflow	Personalized modeling; validation studies
AI-driven predictive surgical planning	Predictive analytics, ML models	Translational evidence	Moderate	Limited clinical use	Improved planning accuracy (early evidence)	Limited dataset diversity; overfitting risk	Clinical validation; reproducibility	Prospective validation trials
Image-guided targeted drug delivery	Imaging + pharmacology integration	Translational evidence	Low–Moderate	Early clinical trials	Improved targeting precision (experimental)	Off-target effects; delivery inefficiency	Drug-device co-regulation	Smart targeting systems
Bio-responsive surgical systems	Biosensors + adaptive control	Conceptual framework	Low	Not implemented	Not established	Requires real-time sensing integration	Safety and validation challenges	Closed-loop therapeutic validation
Smart surgical instrumentation	Sensor-integrated tools	Translational evidence	Moderate	Prototype and early clinical use	Enhanced feedback and precision	Cost; integration complexity	Device approval pathways	Scalable sensor integration

This multidimensional stratification highlights a critical disconnect between technological innovation and clinical translation. While robotic-assisted surgery has achieved widespread clinical adoption, most emerging technologies including AI-driven cognition, pharmacological integration, and bio-responsive systems remain within translational or conceptual domains. Importantly, technical feasibility frequently precedes demonstration of meaningful clinical benefit, emphasizing the need for rigorous validation frameworks. Furthermore, regulatory complexity, interoperability challenges, and variability in real-world performance represent substantial barriers to implementation. This analysis underscores the importance of prioritizing clinically relevant endpoints, standardized evaluation methodologies, and interdisciplinary collaboration to facilitate the safe and effective integration of next-generation intelligent surgical systems into routine practice.

The first surgical robots introduced telemanipulation, decoupling surgeon movement from instruments to improve dexterity and vision. Over the last decade, robotic platforms have evolved conceptually into systems capable of some autonomous function. For example, autonomous suturing and vascular tasks have been demonstrated in animal models (48, 49). Machine learning enables perceptive robots to interpret tissue feedback. However, all these advancements remain at the conceptual or preclinical level. There are no fully autonomous surgical robots in routine clinical use (50). Notably, early trials combining robotics with intraoperative imaging (e.g., fluorescence guidance) have translated to pilot clinical studies (Translational) but even there, the robot acts mainly as an enhanced tool under surgeon control (51–54).

## 4 System architecture of next-generation robotic surgical ecosystems

The current robotic-assisted surgery systems use multiple system layers which unite mechanical operation with sensory data collection and computational processing and therapeutic control functions in single surgical platforms (55, 56). The development of robotic kinematics and materials science has led to the creation of basic platforms which include modular arms and miniaturized effectors and soft robotic structures and magnetically guided components (57, 58). Medical technological advancements now enable better safety measures while they protect tissues from harm and let doctors access regions which were previously unreachable. The system may allow users to deploy micro-devices and injectable therapeutics and bioactive scaffolds through its physical interface which provides exact spatial control.

The mechanical structure supports an advanced sensory system which becomes more complex at higher levels. The field of high-definition stereoscopic optics now combines with hyperspectral imaging and fluorescence-guided surgery and optical coherence tomography and spectroscopic methods which enable real-time detection of biochemical and metabolic states (59–61). Medical personnel can use these systems to detect cancerous tissue from normal tissue while they observe tissue blood flow and monitor both inflammatory reactions and drug tracer movement. The combination of sensory modalities with robotic systems may allow surgeons to conduct operations through data analysis instead of depending on visual feedback.

The computational layer of next-generation systems integrates sensory inputs using machine learning algorithms, digital twin frameworks, and predictive modeling techniques (62, 63). These tools enable robotic platforms to predict tissue responses and create surgical paths and predict the effects of surgical actions on body functions. The models of computational pharmacology help surgeons choose better surgical options because they show how drugs move through tissues and how they interact with receptors and produce treatment outcomes. The combination of surgical data with pharmacological modeling represents a major change from typical medical practices because it makes therapeutic information an essential part of surgical procedures.

**Figure 1:**
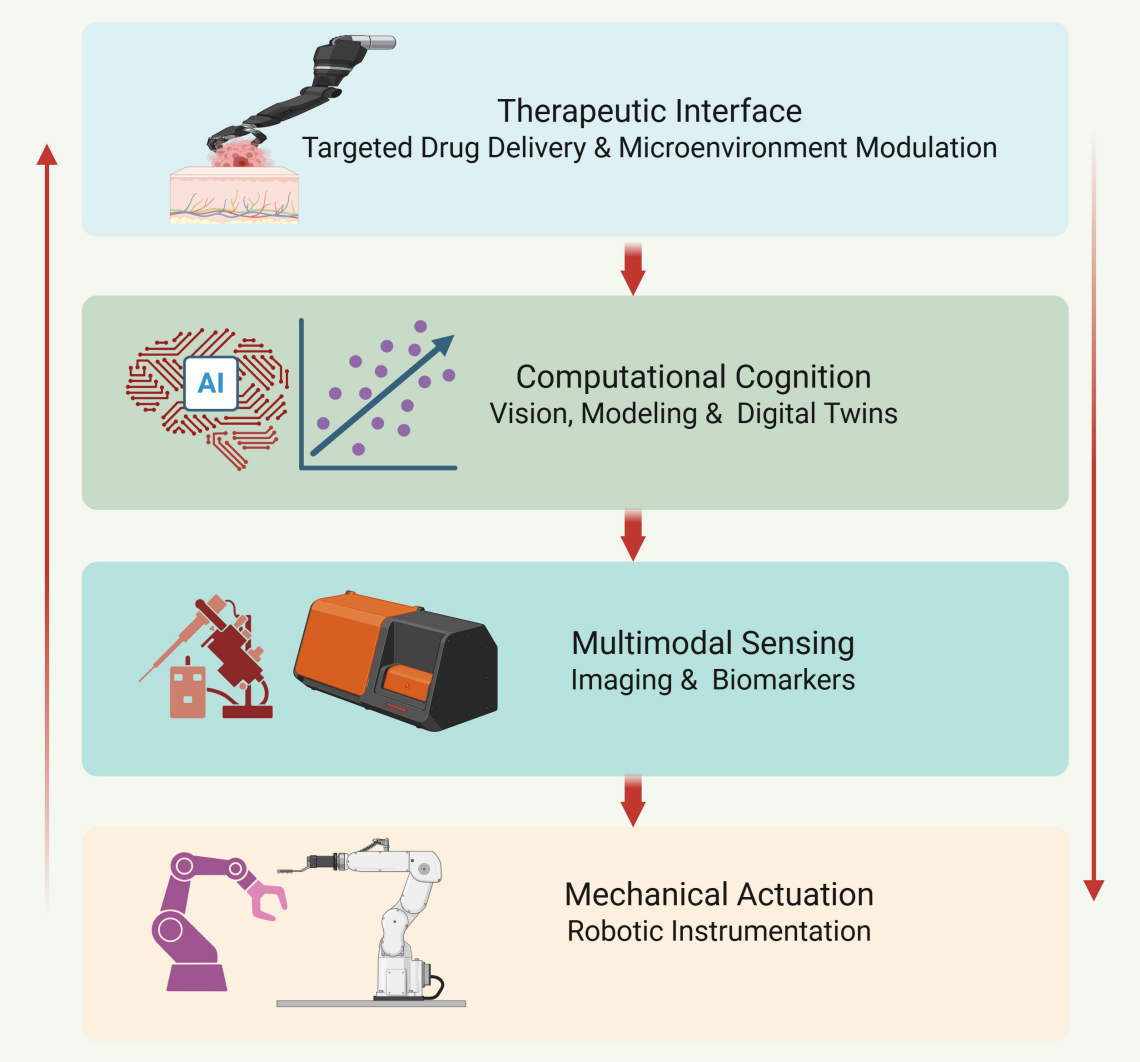
Layered system architecture of next-generation intelligent robotic-assisted surgical ecosystems. This figure was created using BioRender.com (Wali, A. F., 2026, https://BioRender.com/86hr60b) by one of the co-authors using a personal BioRender license. This figure illustrates the hierarchical architecture of next-generation intelligent robotic-assisted surgical platforms. The foundational mechanical actuation layer comprises robotic instrumentation and modular kinematics. Above this, multimodal sensing integrates imaging modalities and biomarker detection for real-time biological assessment. The computational cognition layer incorporates AI-driven vision systems, predictive modeling, and digital twin frameworks to interpret sensory data and forecast tissue and pharmacological responses. The therapeutic interface represents the topmost layer, enabling targeted drug delivery, microenvironment modulation, and adaptive therapeutic execution. Together, these layers establish the operating room as a cyber-physical translational ecosystem capable of real-time biologically responsive intervention. This figure represents a conceptual systems-level architecture intended to illustrate integration pathways and should not be interpreted as a fully validated clinical workflow.

The therapeutic interface represents the most complex interface which robotic systems produce by building their ecosystem (64). The topmost layer includes robotic drug injectors together with implantable delivery devices and drug-eluting biomaterials and micro-robotic carriers which function as therapeutic payload transporters. These systems function to help with resection operations while they actively control biological processes which include angiogenesis and inflammation and immune system activation and tissue healing. The system design transforms robotic-assisted surgery into a system which supports doctors to perform real-time therapeutic management through the synchronization of surgical procedures with medication administration. next-generation robotic platforms are organized into interconnected mechanical, sensory, computational, and therapeutic layers that collectively enable biologically responsive surgical intervention (**Figure 1**).

The operating room now functions as a translational laboratory because of its complex system design. Medical staff determine surgical options through the combination of anatomical knowledge with functional assessment and molecular biological data analysis. The ecosystems will develop into systems which will support adaptive medical procedures that adjust their treatment methods based on drug response patterns. **Table 2** summarizes the core features, clinical applications, key advantages, and limitations of selected next-generation robotic surgical platforms including Senhance (Asensus Surgical), Versius (CMR Surgical), and Hugo RAS (Medtronic), based on current manufacturer specifications, peer-reviewed literature, and regulatory summaries.

**Table 2:** Emerging next-generation robotic-assisted surgical platforms beyond the da Vinci system.

Platform	Manufacturer	Core features/technology	Clinical applications	Key advantages	Limitations/considerations
Senhance Surgical System (139–141)	Asensus Surgical	Open console with eye-tracking camera control and haptic feedback; reusable laparoscopic instruments; up to 4 independent robotic arms; digital fulcrum integration	General laparoscopic procedures, gynecology, urology, thoracic and abdominal surgeries	Enhanced tactile feedback and familiar laparoscopic motion; reusable instruments reduce per-case cost; ergonomic open cockpit design	Limited instrument articulation compared to wristed systems; lacks extensive long-term comparative trials; fewer advanced energy and stapling instruments available in some configurations
Versius Surgical System (142–144)	CMR Surgical	Modular portable robotic arms; open surgeon console; 3D HD vision; wristed instruments with seven degrees of freedom; bedside unit flexibility	Minimally invasive surgery across general surgery, gynecology, urology, colorectal and thoracic procedures (increasing worldwide adoption)	Modularity supports flexible OR use and easy setup; ergonomic design reduces surgeon fatigue; portable to different ORs	Relatively recent introduction; limited long-term outcome data and standardized training pathways; requires careful port placement to avoid collisions
Hugo RAS System (145–147)	Medtronic	Four independent modular robotic arms; open 3D HD surgeon console; connectivity with digital analytics ecosystem; ergonomic bedside integration	Urologic, gynecologic, colorectal, general surgery (FDA cleared for urologic procedures, global clinical use expanding)	Flexible modular deployment across ORs; integrated analytics and connectivity for surgical insights; ergonomic surgeon experience	Emerging platform with limited large-scale randomized trials; adoption and training still expanding; initial procedural times may be longer in early cases
Common Characteristics of Next-Gen Systems (148–150)	—	Modular architecture; open console ergonomics; reusable instruments; digital integration	Broad minimally invasive surgical indications	Improved cost-efficiency and flexibility; enhanced surgeon ergonomics and teamwork; scalable across specialties	Sparse long-term comparative clinical outcomes; evolving regulatory and training frameworks

Data presented are derived from manufacturer specifications, regulatory summaries, and peer-reviewed literature; regulatory approvals and clinical indications may vary across geographic regions and are subject to ongoing updates.

The emergence of next-generation robotic platforms such as the Senhance Surgical System (Asensus Surgical), the Versius Surgical System (CMR Surgical), and the Hugo RAS System (Medtronic) reflects a structural shift beyond conventional telemanipulation. Unlike earlier master–slave architectures primarily focused on dexterity enhancement, these systems incorporate modular deployment, open-console ergonomics, reusable instrumentation, and digital connectivity. Their architecture facilitates integration with advanced imaging platforms, cloud-based analytics, and data-driven performance monitoring. Importantly, these platforms signal a transition toward interoperable surgical ecosystems capable of supporting artificial intelligence integration, intraoperative analytics, and pharmacologically enabled interventions. While early clinical experience supports feasibility and safety across urologic, gynecologic, and general surgical domains, long-term comparative effectiveness data and standardized credentialing pathways remain under development (65–68). Collectively, these systems represent foundational infrastructure for the evolution of intelligent, adaptive, and therapeutically integrated robotic-assisted surgery.

Intelligent robot-assisted surgery stands as one of the most transformative technological frontiers in oncology (69). In parallel, biomedical science has entered an era defined by highly convergent cyber-physical systems that can interpret and dynamically regulate tumor biology in real time. The biological complexity of cancer underscores the need for adaptive, targeted interventions that control tumor ecosystems rather than merely excise tissue. From a pharmacological perspective, this convergence represents a move toward closed-loop therapy, delivering therapeutic signals directly into tumor and peritumoral compartments while continuously adapting based on tumor response (69). Central to this paradigm is artificial intelligence, which, at a conceptual level, integrates intraoperative data streams to continuously regulate both surgical actions and drug administration.

Early demonstrations of precision therapeutics in surgery have largely been preclinical or in small translational studies (48, 50). Large-scale clinical evidence is still emerging, and current comparative analyses show mixed outcomes. For example, a recent umbrella review (over 1.2 million patients) found that robot-assisted laparoscopic surgery yielded outcomes comparable to conventional laparoscopy for most endpoints (69). In that study, the primary differences were higher cost and longer operative times for robotics, without significant improvements in complication rates. By contrast, targeted reviews in specific cancers (e.g., endometrial cancer) report that robotics can reduce blood loss and conversion rates but may increase operative time and short-term complications (50). These findings highlight that, even at the clinical level, the benefits of robotics may depend on surgical context and surgeon experience.

Next-generation systems are envisioned as layered cyber-physical platforms. At the top, AI-driven modules integrate preoperative imaging, real-time sensors, and predictive tumor models to guide surgery. In the middle, a cognitive core uses computer vision and reinforcement learning to plan instrument trajectories. At the base, robotic arms and advanced instruments execute tasks with sub-millimeter precision. For instance, conceptual designs propose embedding drug reservoirs in robotic tools for on-demand delivery. These ideas remain hypothetical (Conceptual) but are supported by preclinical prototypes (48, 49). The key challenge is engineering a fully closed loop of sensing, computation, and actuation under operative constraints.

## 5 Artificial intelligence as the cognitive core of next-generation robotic-assisted surgery

Modern robotic-assisted surgery now depends on artificial intelligence because it has developed from its initial role as an analytical tool into its current position as the system’s central cognitive element (70, 71). Healthcare platforms, today use machine learning alongside deep neural networks and probabilistic modeling to study surgical environments while they forecast patient physiological responses and choose appropriate surgical methods during medical procedures (72–74). The AI-enabled platforms of today operate differently from previous robotic systems because they use their ability to process multiple data streams which include high-resolution images and instrument telemetry and biosensor outputs and patient-specific clinical data (75–77).

The training of computer vision systems with thousands of surgical videos that have been annotated may allow them to automatically detect anatomical landmarks and tissue planes and vascular structures and pathological signatures. The system provides real-time navigation and risk assessment and error correction functions. Deep learning models now demonstrate the ability to predict bleeding events and detect dangerous dissection areas and determine the best instrument paths (26, 78). The development of these systems will enable robotic platforms to evolve from command-following devices into surgical assistants which will improve surgeon mental performance during procedures.

Modern artificial intelligence systems perform intraoperative analytics through advanced methods which enable better results than human sensory abilities (79–81). Robots learn new procedural methods through reinforcement learning and predictive modeling which they use to improve their operations based on continuous feedback reception. The systems use learning processes to discover the best surgical methods and stitching methods and power usage strategies by analyzing surgical information from databases and virtual operating environments. Digital twin technology extends this paradigm through its ability to generate computational models which represent patient anatomy and physiology for surgeons to practice procedures and predict drug distribution and tissue reactions before and during surgical operations (82).

**Figure 2:**
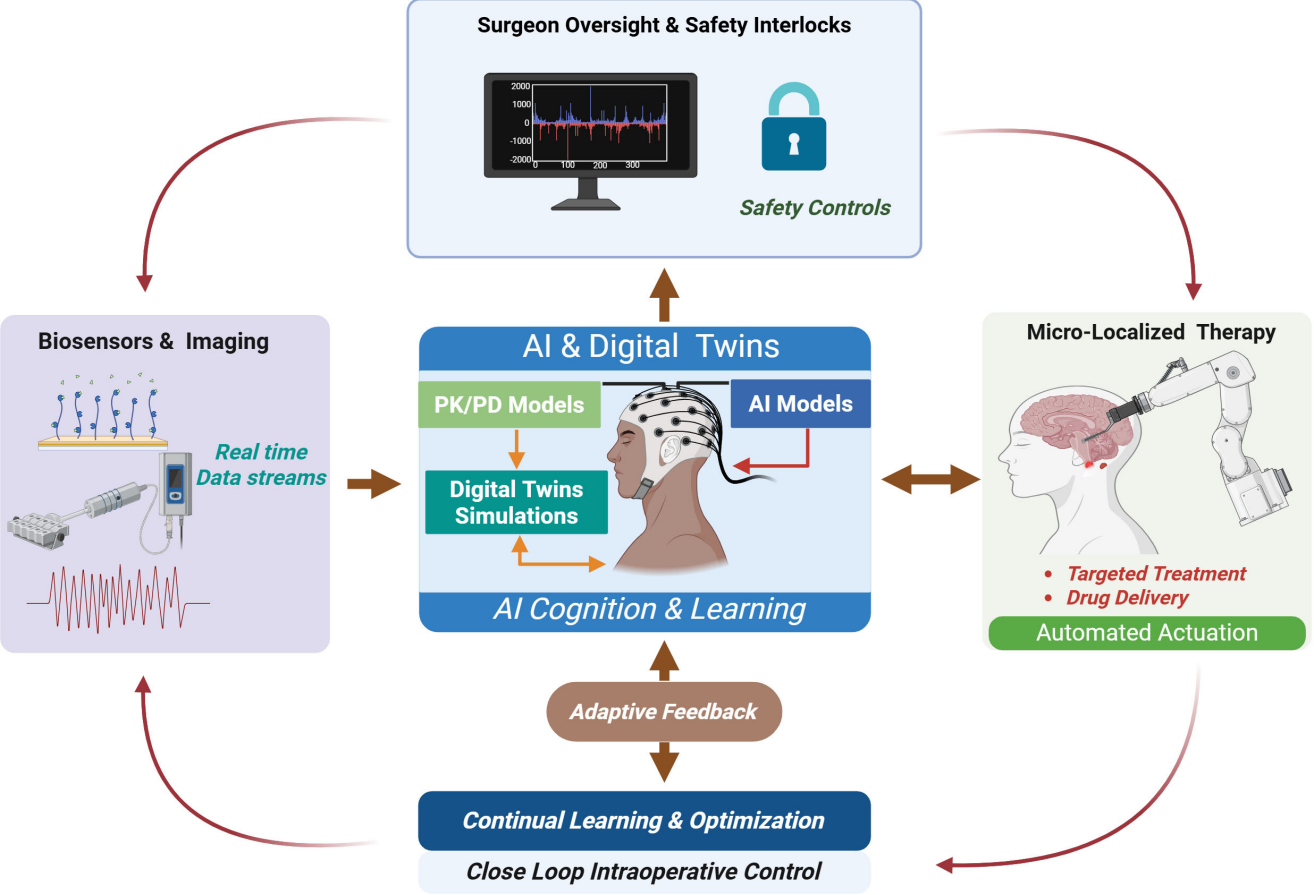
Closed-loop intelligent robotic-assisted surgical ecosystem integrating AI, digital twins, and adaptive therapeutic control. This figure was created using BioRender.com (Wali, A. F., 2026, https://BioRender.com/86hr60b) by one of the co-authors using a personal BioRender license. This figure illustrates the conceptual architecture of an intelligent therapeutic robotic-assisted surgery platform. Real-time multimodal biosensing and imaging streams feed computational artificial intelligence (AI) models and pharmacokinetic–pharmacodynamic (PK/PD) frameworks. These inputs generate patient-specific digital twin simulations that predict tissue response, drug distribution, and biological dynamics. The AI cognition layer continuously refines intraoperative decision-making through adaptive feedback mechanisms. Micro-localized robotic actuation has the potential to enable spatially precise drug delivery and targeted tissue intervention under surgeon oversight and embedded safety interlocks. The system operates as a closed-loop intraoperative control framework that synchronizes sensing, modeling, cognition, and therapeutic execution.

Artificial intelligence assists pharmacological treatment by creating a connection between surgical procedures and drug response (83). AI systems use pharmacokinetic-pharmacodynamic models together with intraoperative biosensing to monitor drug distribution and receptor binding and biological responses which happen in specific tissue microenvironments (84–86). The system may allow robotic platforms to make immediate drug delivery adjustments through real-time monitoring of physiological changes which facilitates them to optimize drug amounts and release patterns and delivery locations. AI-guided robotic systems in oncology use dynamic mapping to detect tumor heterogeneity which aids them to optimize the delivery of treatments inside tumors (16). AI models in metabolic and regenerative applications use their predictive abilities to forecast three main processes which include inflammatory cascades and insulin sensitivity shifts and tissue repair dynamics for developing specific pharmacological treatments (45, 87). Next-generation robotic-assisted surgery operates within a closed-loop ecosystem where real-time biosensing, AI-driven digital twin simulations, and PK/PD modeling converge to enable adaptive, micro-localized therapeutic control under surgeon oversight (**Figure 2**).

Medical professionals can now move from their current procedure-based work to new intelligence-based operative medical practices through the advancement of cognitive robotic-assisted surgery. Artificial intelligence operates as an extension of surgical expertise which facilitates medical professionals to analyze data and make biological predictions and optimize treatments beyond what humans can accomplish. The development of AI will continue to unite robotics with pharmacology and precision medicine through its role as an integrative system which connects these fields. Pharmacological robotics describes the integration of robotic surgical platforms with targeted drug delivery systems, pharmacokinetic–pharmacodynamic modeling, and real-time therapeutic monitoring, enabling spatially precise and temporally controlled administration of pharmacological agents during surgical procedures. This concept extends robotic-assisted surgery beyond mechanical intervention, positioning it as an active modality for localized, image-guided, and biologically responsive drug delivery within complex tissue environments. Similarly, high-level evidence supporting the use of artificial intelligence for intraoperative decision support remains limited, with most available data positioned at a translational level, reflecting feasibility and early clinical exploration rather than established, large-scale clinical validation.

Critically, while the integration of artificial intelligence, real-time sensing, and pharmacological modulation within robotic surgical systems represents a significant conceptual advancement, the current evidence base remains heterogeneous and predominantly derived from preclinical models, simulation environments, or limited pilot clinical studies. Although several studies report improvements in intraoperative precision, workflow efficiency, or decision-support capabilities, these outcomes are often context-dependent and lack consistent validation across diverse surgical settings and patient populations. Furthermore, methodological variability including differences in algorithm training datasets, absence of standardized performance metrics, and limited external validation poses a substantial barrier to reproducibility and generalizability. Importantly, the distinction between technical feasibility and demonstrable clinical benefit remains insufficiently defined, as many reported advancements have not yet translated into improved long-term patient outcomes such as survival, recurrence rates, or complication reduction. Additionally, issues related to algorithm transparency, regulatory approval pathways, and integration into existing clinical workflows remain unresolved. Taken together, these limitations underscore the necessity for rigorously designed prospective studies, multicenter validation trials, and standardized benchmarking frameworks to establish the true clinical value of these emerging technologies.

## 6 Pharmacology in the era of intelligent robotic-assisted surgery

The integration of pharmacology into robotic-assisted surgery stands as an essential yet not completely understood advancement which will transform the way doctors practice operative medicine. Pharmacotherapy and surgery have existed in separate time and space domains because doctors used to give patients medication through the body before or after their operations while surgical teams performed only mechanical tissue alterations. Robotic-assisted surgery assists doctors to conduct precise image-guided surgical operations which they can operate through pre-programmed commands (88).

Robotic platforms perform their initial medical duty through drug delivery systems which doctors use to execute exact drug delivery procedures during surgical procedures. The deployment of chemotherapeutic agents and biologics and gene therapies and regenerative compounds become possible through robotic injectors and micro-catheter systems which allow precise delivery to specific tissue areas. The exact drug delivery method produces two major effects on drug distribution because it creates high drug concentrations in specific areas while it reduces drug levels throughout the entire body. The oncology field uses robotic-guided intratumoral delivery to place cytotoxic and immunomodulatory agents directly inside tumors which could help solve two major challenges of drug resistance and uneven blood vessel distribution.

Robotic-assisted surgery now provides delivery services which go beyond its first intended use to support theranostic strategies that combine diagnostic and therapeutic medical functions (89). Fluorescently labeled drugs, receptor-targeted probes, and metabolite-sensitive tracers allow robotic systems to visualize pharmacological engagement in real time. The surgical team can monitor drug distribution and target occupancy and immediate biological responses through these technological developments (90). The intraoperative pharmacodynamic data provides a new approach which supports surgeons to conduct therapeutic experiments during surgery instead of performing only structural procedures.

The robotic platforms allow healthcare providers to operate pharmacokinetic and pharmacodynamic modeling systems which help them select surgical procedures (4). Robotic systems will gain the ability to monitor biological responses through biosensors which track tissue oxygenation and pH levels and cytokine patterns and metabolite readings (91, 92). The collected data streams enable developers to build adaptive dosing algorithms which perform real-time therapeutic delivery adjustments to fulfil individual patient requirements during surgical procedures.

The delivery of bioactive compounds through robotic systems in metabolic surgery operations could influence both gut-brain communication and body insulin response. The use of localized pharmacological treatment during cardiovascular interventions helps control thrombosis and inflammation and angiogenesis to enhance the success rate of medical procedures. The exact molecular control of tissue repair becomes possible through robotic implantation of drug-eluting scaffolds and stem-cell-laden biomaterials in regenerative medicine (93, 94). The applications demonstrate that robotic-assisted surgery functions as a pharmacologically enabled medical field which controls intricate therapeutic systems.

Comparative evaluation of existing studies reveals considerable variability in the reported performance and clinical utility of AI-enabled robotic systems. While certain investigations demonstrate enhanced surgical precision, reduced intraoperative variability, and improved ergonomic efficiency, others report marginal or non-significant benefits when compared with conventional robotic-assisted approaches. These discrepancies may be attributed to differences in study design, patient selection, surgical complexity, and the maturity of the underlying technological platforms. For instance, systems trained on highly curated datasets may perform optimally under controlled conditions but exhibit reduced robustness in real-world clinical environments characterized by anatomical variability and unforeseen intraoperative challenges. Additionally, the absence of standardized outcome measures complicates cross-study comparisons, limiting the ability to derive generalized conclusions regarding efficacy. Some studies emphasize improvements in surrogate endpoints, such as procedural time or instrument accuracy, without demonstrating corresponding benefits in clinically meaningful outcomes. This heterogeneity highlights the need for harmonized evaluation frameworks and standardized reporting guidelines. Future comparative studies should prioritize multicenter designs, larger patient cohorts, and integration of clinically relevant endpoints to more accurately define the role of these technologies in routine surgical practice.

One of the studies investigated an ultrasound-guided robotic injection system designed for precise intranodal delivery of dendritic cell vaccines in mouse models, addressing the limitations of manual injection in small lymph nodes (95). The robotic platform demonstrated superior targeting accuracy (~285 μm) compared to conventional manual techniques and was validated in both phantom and *in vivo* settings, highlighting improved precision and control. These findings emphasize the role of robotic-assisted, image-guided micro-interventions in enhancing procedural accuracy in preclinical research. These proof-of-concept experiments establish feasibility (Translational), but human trials have not yet been reported, and clinical evidence remains sparse despite emerging conceptual and preclinical advancements.

## 7 Robotic-assisted surgery in oncology as a platform for precision therapeutics

The practice of oncologic surgery has always focused on removing tumors completely while protecting surrounding tissues, but its current methods face two major restrictions which stem from human vision limitations and surgeon hand accuracy. Robotic-assisted surgery assists oncologic practice to benefit from three main advantages which include tumor access to hard-to-reach areas and improved visual control and superior surgical precision. The most significant change that surgery can make happens when it develops into a precise treatment system which uses molecular tests to find specific drugs for patients.

**Figure 3:**
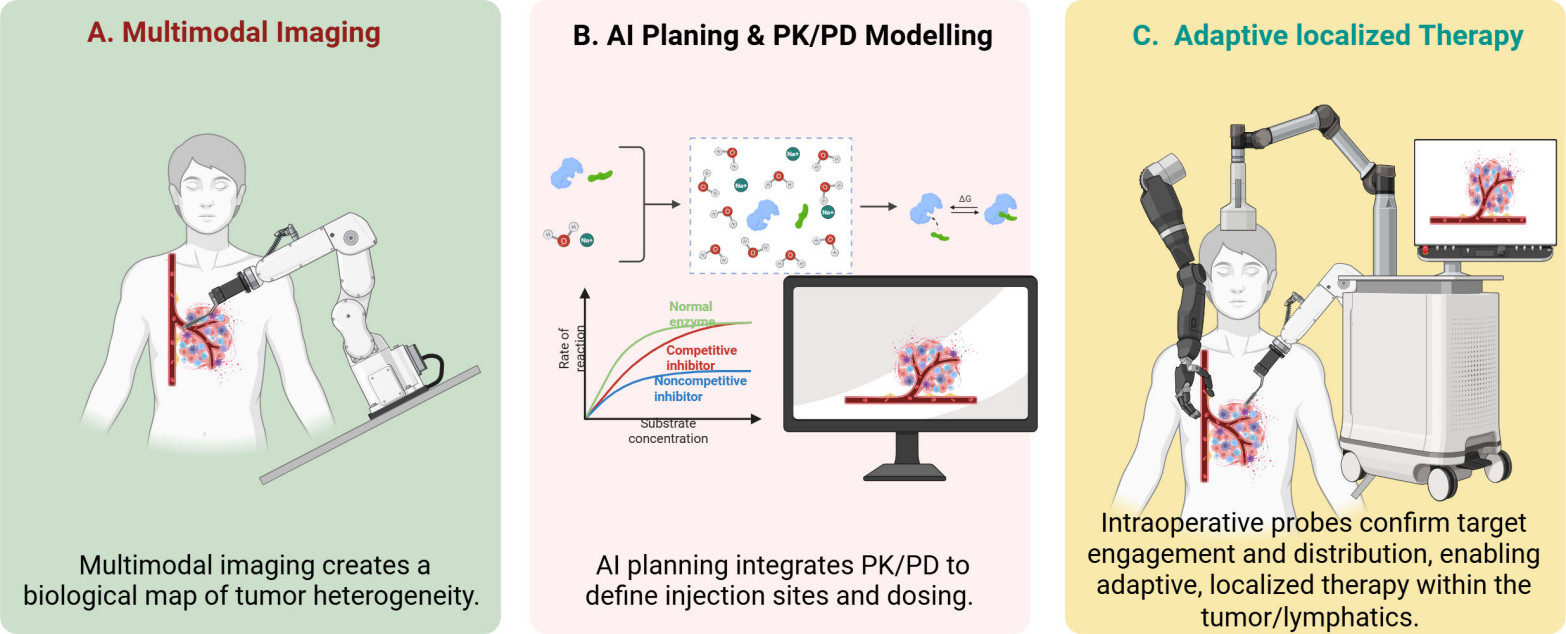
AI-guided multimodal imaging and adaptive localized therapeutic delivery in oncologic robotic-assisted surgery. This figure was created using BioRender.com (Wali, A. F., 2026, https://BioRender.com/86hr60b) by one of the co-authors using a personal BioRender license. This figure demonstrates the translational workflow of intelligent oncologic robotic-assisted surgery. **(A)** Multimodal imaging generates a spatially resolved biological map of tumor heterogeneity. **(B)** Artificial intelligence integrates imaging data with pharmacokinetic–pharmacodynamic (PK/PD) modeling to determine optimal injection sites, dosing strategies, and therapeutic distribution profiles. **(C)** Intraoperative probes confirm target engagement and drug localization, enabling adaptive, localized therapy within tumor and lymphatic microenvironments. The framework illustrates how molecular imaging, predictive modeling, and robotic actuation converge to transform surgical oncology into a spatially controlled therapeutic platform.

The current robotic systems use fluorescence imaging and hyperspectral analysis and spectroscopic sensors which enable them to identify cancerous tissues through their molecular characteristics (96–98). These technologies enable doctors to perform real-time tumor margin definition and micro metastasis detection and biologically aggressive tumor area identification (99). The integration of AI-based tissue classification with robotic systems may allow doctors to create intraoperative tumor maps which display the distribution of different metabolic and vascular and receptor characteristics in the tissue (10, 100). **Figure 3** depicts the integration of multimodal tumor mapping, AI-driven PK/PD planning, and intraoperative validation that supports adaptive, localized oncologic therapy within heterogeneous tumor microenvironments.

Molecular visualization technology may allow doctors to perform surgical procedures through a therapeutic approach which they can control during the operation. Robotic systems enable the precise delivery of chemotherapeutics and immunomodulators and gene therapies to tumor sites and lymphatic pathways and remaining cancer cells (101). The delivery method of localized administration changes how drugs affect the body because it directs medications to specific biological areas which reduces the risk of side effects throughout the body. The new approach has the potential to enable surgical oncology to use drug therapy principles which convert surgical procedures into targeted drug delivery methods.

Robotic oncology facilitates theranostic strategies through which doctors can attach therapeutic agents to imaging probes which enable them to visualize and treat patients at the same time (102–104). The methods allow surgeons to check how treatments penetrate tissues while confirming their effects on targets so they can right away modify their treatment plans. Surgical patients with cancer now have access to monitor their pharmacodynamic responses which supports them to receive biological feedback about their treatment.

The present medical environment facilitates surgeons to conduct regional oncologic treatments through hyperthermic intraperitoneal chemotherapy and targeted lymphatic drug delivery and localized immunotherapeutic modulation using robotic systems (101, 105). The treatment approaches demonstrate how surgical methods join forces with pharmaceutical approaches to create a single approach for cancer treatment. AI analytics will enable robotic systems to use predictive capabilities which will help them identify tumor response patterns and select the best treatment approaches and make instant changes to surgical and pharmacological treatment protocols. Oncologic robotic-assisted surgery has evolved from its original purpose as a precise mechanical system into a platform which has the potential to enable precise cancer treatment through the combination of molecular diagnostic tools and targeted medical treatments and adaptive intelligence systems (4, 106–108).

Recent clinical evidence further supports the evolving role of robotic-assisted oncology, while also highlighting important limitations. In a small retrospective study by Petric et al., robotic-assisted simultaneous resection of colorectal cancer and liver metastases demonstrated feasible and safe short-term outcomes comparable to open surgery; however, the limited sample size and relatively high complication rate restrict broader generalizability (109). In contrast, a large multicenter analysis by Xia et al. showed that robot-assisted liver resection was associated with lower complication rates, reduced advanced-stage recurrence, and improved quality-adjusted survival compared to open approaches, although longer operative times and higher costs remain challenges (110). Collectively, these findings indicate that while robotic-assisted surgery has established clinical feasibility and perioperative benefits, its integration as a platform for precision therapeutics remains at a predominantly translational stage, with limited high-level evidence supporting long-term oncologic superiority.

## 8 Robotic-assisted surgery in metabolic and regenerative medicine

Modern medicine faces two of its most complicated medical issues which include metabolic disease and tissue degeneration because they involve complete body system problems and ongoing inflammation and the inability of tissues to repair themselves. Robotic-assisted surgery has proven to be highly beneficial for bariatric and metabolic procedures because it facilitates better control of anatomy and leads to fewer complications during and after surgery (111, 112). Scientists use advanced robotic systems for biological metabolic control and tissue regeneration research through their development of complex robotic systems.

The precise robotic techniques used in metabolic surgery enable doctors to perform detailed operations on the gastrointestinal and endocrine systems which control blood sugar levels and fat metabolism and hormone-based signaling pathways. Robotic systems now enable the delivery of bioactive compounds which enable specific gut-brain signaling and treatment of adipose inflammation and pancreatic regeneration. The pharmacological aspect of incretin analogs and anti-inflammatory agents and cellular therapies which doctors use to treat specific tissues in the body creates an additional therapeutic effect which goes past the physical modifications made during surgery.

Medical science has achieved its most advanced translation stage because researchers developed regenerative robotic-assisted surgery. The use of robotic systems has become common for precise implantation of drug-eluting scaffolds and stem-cell-laden matrices and bio responsive hydrogels at the microscale level (93). These constructs release growth factors and immunomodulators and morphogens through controlled spatial and temporal mechanisms which direct tissue repair processes. The robotic system achieves precise placement of cells which leads to maximum regenerative effects in areas with ischemic myocardium and neural injury zones and musculoskeletal defects and hepatic lesions (113–115).

The pharmacological aspects of regenerative robotic-assisted surgery combine drug delivery systems with biomaterials knowledge and cellular therapy methods (116, 117). Robotic systems can use materials which react to enzymatic enzymes and pH changes and inflammatory substances to deliver therapeutic agents when needed. The AI-enabled platforms will use their monitoring systems to track regenerative biomarkers which will enable them to perform therapeutic adjustments through biological signal feedback during surgery and after implantation.

The integration of robotics with regenerative pharmacology may allow surgery to become a biological reconstruction tool which goes beyond its current role of performing basic tissue substitution. The development of stem-cell engineering and exosome therapeutics and smart biomaterials will require robotic systems to perform individualized regenerative medical treatments.

Outside oncology, robotic surgery is expanding into metabolic procedures (e.g., bariatrics) and regenerative medicine (e.g., pancreatic islet transplants) (118–121). These applications provide analogies: for instance, AI-guided robots have delivered insulin-producing cells with improved engraftment (122). This suggests potential for similar cancer immunotherapy deliveries. However, direct cancer-specific examples are not yet published. Thus, we note the conceptual parallels (Conceptual) but emphasize that oncology-specific translational data are still minimal.

## 9 Regulatory, translational, and pharmacological future frameworks

The development of robotic-assisted surgery systems which use intelligence and pharmacological integration requires new regulatory methods that extend past existing medical device inspection procedures (123, 124). These platforms serve as mechanical instruments while also functioning as diagnostic systems and drug-delivery devices and decision-support technologies. The combination of these fields generates problems for existing regulatory frameworks because they previously maintained distinct separation between medical devices and pharmaceuticals and digital health systems.

The new regulatory framework needs to establish safety protocols for equipment while making algorithm operations transparent during machine learning processes and drug surveillance and cyber defense activities (23, 125, 126). Robotic systems which operate independently or with partial autonomy for therapeutic treatment need complete validation to achieve reliable results in different types of patients. The delivery of pharmacological agents through robotic platforms requires regulatory agencies to establish new approval procedures which combine medical device evaluation with drug testing protocols for pharmacokinetic studies and tissue distribution analysis and biological effect assessments over time.

The successful clinical translation of intelligent therapeutic robotic systems requires a multi-stage validation pipeline encompassing *in vitro* modeling, preclinical animal studies, early-phase human trials, and post-market surveillance. Critical evaluation parameters include safety, reproducibility, pharmacokinetic consistency, algorithmic transparency, and real-time system reliability. Importantly, regulatory approval pathways must evolve to accommodate hybrid drug-device-AI platforms, necessitating integrated evaluation frameworks that simultaneously address pharmacological efficacy, device safety, and algorithmic performance. The process of multidisciplinary collaboration needs to extend its reach into translation work. The development of platforms needs engineers to work with pharmacologists and surgeons and data scientists and regulatory experts who must unite technological advancements with biological complexities and medical functionality. The development of preclinical models which link disease-related pharmacodynamic responses to robotic performance indicators will serve as essential elements for medical safety evaluation of these devices.

Robotic-assisted surgery will become an essential part of precision therapeutics which will determine the direction of future pharmacological practices. Doctors will employ omics technologies together with digital twins and AI-based modeling systems to forecast drug responses before surgery and to adjust medical protocols during surgical procedures. The framework shows robotic systems which operate as therapeutic platforms that maintain biological equilibrium through their mechanical and molecular therapeutic methods.

## 10 Advancing cognitive and semi-autonomous therapeutic surgery

The development of robotic-assisted surgery systems will concentrate on building systems which merge human thinking with machine execution to handle complex decision-making tasks. The development of artificial intelligence technology has led to three significant advancements because of its ability to perform automated suturing and its autonomous camera control system and its tissue classification capabilities (127, 128). Robotic systems will gain new capabilities which will allow them to analyze anatomical and physiological and molecular data streams to generate operation plans for certain conditions.

The cognitive framework requires pharmacological intelligence to function as its main structural element. Robotic systems can use predictive models which forecast drug responses and tissue reactions and individual patient differences to create control systems which predict treatment requirements and adjust their operations based on these predictions (5, 129). The system uses algorithms to predict inflammatory responses based on surgical trauma evaluation which facilitates prompt administration of immunomodulatory medications (130, 131). Robotic systems can model tumor heterogeneity during surgical procedures while they optimize drug delivery to specific malignant areas of the tumor (132, 133).

The integration of autonomous systems with pharmaceuticals may allow developers to create surgical treatment systems which function through closed feedback systems. The systems operate robotic platforms which track biological data and identify system changes to perform treatment procedures. The feedback-controlled interventions allow surgeons to function as living system regulators instead of executing separate mechanical procedures. The method follows the principles of operative medicine which matches with current developments in cybernetics and biology that describe health as an adaptive process of continuous modulation.

Cognitive surgical systems refer to next-generation robotic platforms augmented with artificial intelligence and data-driven computational frameworks that enable perception, learning, decision support, and predictive modeling within the surgical environment. These systems are designed to interpret multimodal intraoperative data including imaging, sensor outputs, and patient-specific information to assist or augment surgical decision-making, optimize procedural strategies, and potentially support semi-autonomous task execution under human supervision. The development of cognitive surgical systems encounters major challenges which impact medical translation systems and need changes to ethical and regulatory frameworks (134, 135). The integration of artificial intelligence and pharmacological decision-making into robotic systems raises significant ethical challenges, including algorithmic accountability, transparency in decision pathways, informed consent in semi-autonomous systems, and responsibility attribution in the event of adverse outcomes. Establishing robust ethical frameworks will be essential to ensure patient safety, maintain clinician oversight, and support responsible innovation. The combination of drug delivery systems with autonomous decision systems requires developers to create new systems which will handle pharmacovigilance and liability and clinical governance. Medical devices which perform surgical robot functions for therapeutic purposes create difficulties in determining their status between medical equipment and drug delivery systems because they do not fit into current regulatory categories (136–138). The solution to these problems needs teams which unite engineers with surgeons and pharmacologists and ethicists and policy makers.

The development of cognitive therapeutic surgery remains active although its complex nature presents challenges to its advancement. The development of robotic systems which possess intelligence and biological perception and therapeutic abilities will create new limits for operative medical practices. The future of surgery will bring an adaptive therapeutic approach which uses data to create individualized treatments that work together with pharmacological science.

As the field of intelligent robotic-assisted surgery continues to evolve at an unprecedented pace, it is essential to interpret its progress with measured scientific caution. While the technological advancements described in this review are undeniably promising, much of the current evidence remains rooted in early developmental and validation stages. Most reported findings originate from controlled experimental environments, simulation-based platforms, or limited-scale feasibility studies, rather than from large, multicenter clinical trials that define true clinical maturity. This gap between innovation and validation is further compounded by heterogeneity in study designs, variability in outcome reporting, and the absence of standardized evaluation frameworks, all of which collectively limit the generalizability and real-world applicability of these emerging technologies. In addition, the scarcity of long-term follow-up data makes it difficult to fully assess durability, safety, and sustained therapeutic benefit. Taken together, these challenges underscore a critical need for rigorously designed prospective studies and robust, regulatory-aligned validation pathways to bridge the translational divide.

Within this context, the present review itself reflects the evolving nature of the field it seeks to synthesize. By design, it draws upon a diverse body of literature encompassing clinical applications, preclinical investigations, and forward-looking conceptual frameworks. Although careful efforts were made to prioritize high-quality and methodologically sound evidence, the rapidly advancing landscape of robotic-assisted surgery and artificial intelligence inevitably introduces temporal gaps between technological innovation and clinical validation. As a result, several of the concepts discussed particularly those relating to closed-loop therapeutic systems and pharmacologically integrated robotic platforms remain, at present, largely speculative and supported predominantly by early-stage experimental data. Recognizing these limitations is essential, not as a constraint, but as a reflection of a field in transition. It calls for cautious interpretation of current evidence while simultaneously emphasizing the urgent need for well-structured, multicenter, and regulatory-grade studies that can transform these conceptual advances into clinically validated realities.

## 11 Conclusion

The convergence of robotic-assisted surgery, artificial intelligence, and pharmacological integration signals a paradigm shift in precision oncology, redefining the operating room from a passive procedural setting into a potentially intelligent, data-responsive therapeutic interface. This evolving landscape reflects not merely technological augmentation, but a deeper conceptual transition toward integrating surgical intervention with real-time biological insight and adaptive therapeutic modulation.

However, this transformation is characterized by a clear gradient of technological maturity that must be critically acknowledged. While robotic-assisted surgical platforms are firmly established in clinical practice, demonstrating reproducible improvements in precision, ergonomics, and perioperative outcomes, the integration of artificial intelligence, molecular sensing, and pharmacologically guided interventions remains largely within translational and preclinical domains. Current evidence supports the technical feasibility of these systems particularly in image-guided decision support, localized drug delivery, and intraoperative data acquisition but their translation into consistent, generalizable clinical benefit is constrained by methodological heterogeneity, limited prospective validation, and challenges in real-time multimodal data integration.

More advanced constructs, including closed-loop surgical systems and adaptive therapeutic robotics, represent an emerging conceptual frontier. These frameworks propose a shift from static, episodic intervention toward continuous, feedback-driven regulation of tumor biology, thereby aligning surgical practice with principles of systems medicine. Nevertheless, such models remain in early-stage development, supported primarily by proof-of-concept studies and simulation-based environments. Substantial barriers including data fidelity, algorithmic robustness, regulatory oversight, safety validation, and ethical considerations must be systematically addressed before clinical realization can be achieved. Importantly, the distinction between technological plausibility and clinically validated efficacy remains a critical determinant of responsible innovation in this field.

Looking ahead, the future of intelligent surgical oncology will depend on the seamless integration of multidisciplinary expertise spanning surgery, computational sciences, pharmacology, and bioengineering. Advances in systems biology, real-time biosensing, and predictive analytics hold the potential to further enhance intraoperative decision-making and enable truly personalized therapeutic strategies. However, sustained progress will require not only technological innovation but also commitment to methodological rigor, ethical accountability, and patient-centered outcomes.

Ultimately, while the vision of a fully integrated, biologically responsive, and adaptive surgical ecosystem remains an emerging paradigm, its successful realization will hinge on a careful balance between innovation and evidence. A disciplined, critically appraised, and translationally grounded approach will be essential to ensure that these advancements move beyond conceptual promise to deliver meaningful and measurable impact in oncologic care.
